# Structure and Phase Composition of WNb Alloy Formed by the Impact of Compression Plasma Flows

**DOI:** 10.3390/ma16124445

**Published:** 2023-06-17

**Authors:** Azamat Ryskulov, Vitaliy Shymanski, Vladimir Uglov, Igor Ivanov, Valiantsin Astashynski, Bauyrzhan Amanzhulov, Anton Kuzmitski, Alisher Kurakhmedov, Andrei Filipp, Yerulan Ungarbayev, Mikhail Koloberdin

**Affiliations:** 1 Institute of Nuclear Physics, Almaty 050032, Kazakhstan; i.ivanov@inp.kz (I.I.);; 2 Department of Solid State Physics, Belarusian State University, 220030 Minsk, Belarus; shymanskiv@mail.ru (V.S.); uglov@bsu.by (V.U.); 3 Engineering Profile Laboratory, L.N. Gumilyov Eurasian National University, Astana 010008, Kazakhstan; 4 A.V Luikov Heat and Mass Transfer Institute of National Academy of Science of Belarus, 220072 Minsk, Belarus; 5 Department of Nuclear Physics, New Materials and Technologies, Physical-Technical Faculty, L.N. Gumilyov Eurasian National University, Astana 010008, Kazakhstan; 6 Department of General Physics, Belarusian State University, 220030 Minsk, Belarus

**Keywords:** tungsten, niobium, WNb alloy, plasma mixing, compression plasma flow, X-ray diffraction, phase composition, solid solution, structure

## Abstract

The results of a tungsten–niobium alloy synthesis by the impact of pulsed compression plasma flows are presented. Tungsten plates with a 2 μm thin niobium coating were treated with dense compression plasma flows generated by a quasi-stationary plasma accelerator. The plasma flow with an absorbed energy density of 35–70 J/cm^2^ and pulse duration of 100 μs melted the niobium coating and a part of the tungsten substrate, which caused liquid-phase mixing and WNb alloy synthesis. Simulation of the temperature distribution in the top layer of the tungsten after the plasma treatment proved the formation of the melted state. Scanning electron microscopy (SEM) and X-ray diffraction (XRD) were used to determine the structure and phase composition. The thickness of the WNb alloy was 10–20 μm and a W(Nb) bcc solid solution was found.

## 1. Introduction

Tungsten heavy alloys (WHAs) with high melting points, thermal conductivity, high strength and radiation stability are widely considered promising materials for high-temperature applications. In particular, the field of new fusion reactor construction is interested in the use of WHAs for plasma-facing component production or as coatings. Having a high displacement energy under the ion and neutron irradiation, tungsten-based materials have high erosion resistance that allows them to be used as plasma-facing materials. The strong requirements for the first-wall materials, including a high melting point, high strength and radiation stability of the structure, sufficiently limit the number of potential metals and alloys. The operation of the thermonuclear chamber with a plasma-localized area surrounded by a magnetic field implies a high heat flux acting on the wall [1,2,3,4,5]. It provides high rates of heating and cooling of the surface that result in mechanical stress and destruction. The improvement in the thermo-mechanical properties of tungsten is still a crucial problem. Pure tungsten has a ductile-to-brittle transition temperature (DBTT) higher than that for steels and other metals. It is widely accepted that there are two main factors influencing the DBTT: (i) an intrinsic lack of close-packed planes in the BCC structure and (ii) the poor cohesion of grain boundaries [6,7].

The limitations of pure tungsten application can be overcome by alloying. Traditional methods of metal alloys’ synthesis based on heating above the melting point and liquid-phase mixing have a significant limitation for refractory metals since their molten state requires molds with even higher melting points. The methods of tungsten-based alloys’ production usually include powder technologies where fine metal particles are compacted together at high temperatures [8,9], giving a wide range of the final compositions of the alloys. However, the formed alloys have a rather high porosity and their radiation resistance is rather poor. The alloys with a uniform structure have a higher range of practical application. As the thin subsurface layer plays an important role in the erosion processes, radiation effects, and mechanical and wear resistance, it is suggested to make an alloy just in the subsurface layer of any other metal. The alloy will have the physical properties of the base metal but the surface properties will be modified. The synthesis of the metal alloys on the surfaces was implemented by means of high-energy pulsed plasma exposure [10,11]. The plasma streams generated by quasi-stationary plasma accelerators were used to melt the “substrate/coating” samples. This surface treatment gave a combination of the high energy density required to melt the metals and the long pulse duration that ensured efficient mixing of the two molten layers. Compression plasma flows have demonstrated their effectiveness for the synthesis of Ti-, Zr-, Al- and Fe-based alloys. The modification of tungsten’s surface as well as WTi alloy formation by means of compression plasma flows was demonstrated [12].

As an alloying element for tungsten, niobium has a high melting point, high corrosion resistance and low thermal neutron absorption cross-section [13,14]. Alloying of tungsten with niobium shows good potential since it forms an isomorphous system with tungsten and does not form any eutectic or brittle intermetallic compounds. Moreover, in the case of oxidation, niobium oxides are more stable than tungsten oxides [15], thus providing protection of tungsten’s surface by preferentially forming niobium oxides. The present work used the pulsed action of compressive plasma flows on the tungsten with preliminary deposited niobium coating to form a tungsten–niobium (WNb) alloy. This work studied the structure and phases of the WNb alloy with different Nb concentrations formed by the impact of compression plasma flows.

## 2. Materials and Methods

Tungsten samples as plates with dimensions of 10 mm × 10 mm and thickness of 2 mm were used. The plates were made as bulk tungsten alloy of high purity (W concentration 99.6 at.%, Al, Ti and Fe—0.4 at.%). After surface cleaning, the Nb coating was formed by arc vacuum deposition in an inert Ar atmosphere. The thickness of the Nb coating was 2 μm. The Nb/W “coating/substrate” system was subjected to the compression plasma flows (CPF) generated in a magnetoplasma compressor of compact geometry. The plasma stream was formed in a “residual gas” mode in which a pre-evacuated vacuum chamber was filled with nitrogen up to the pressure of 3 Torr. The time of stable compression flow of 100 μs was considered as the pulse duration, after which the plasma stream was unstable and started to dissipate. The samples were placed inside the chamber in a vertical position 10–6 cm from the plasma source. The plasma stream was produced during the gas discharge between electrodes with the voltage of 4.0 kV. The structure and phase composition of the materials modified by the plasma flows mainly depend on the heat energy transferred to the surface layer, and the parameters used provided the absorbed energy density from 35 to 70 J/cm^2^ [12]. The CPF influence on the Nb/W samples was by three and five pulses 20–30 s apart. The detailed description of the CPF device operation is given elsewhere [16].

The varied absorbed energy density of the plasma impact facilitated forming the WNb alloys with different Nb concentrations and the composition of the alloys was studied by energy-dispersion X-ray microanalysis (EDX) on an Oxford MaxN analyser with an accelerating voltage of 20 kV on a LEO 1455 VP scanning electron microscope (SEM). Both secondary electrons mode (SE) and back-scattered electrons mode (BSE) were used. This method was used for both surface and cross-section studies. The error of the tungsten and niobium contents was not more than 0.5%. The line-scan approach was used to obtain Nb concentration profile in a depth of the samples The phases of the modified layers in the WNb alloys were determined by X-ray diffraction (XRD) using an Ultima IV RIGAKU diffractometer with Bragg–Brentano geometry and Cu Kα radiation (wavelength 0.154178 nm). The lattice parameters of the phases were calculated with the diffraction peaks at the highest Bragg’s angle, so the error in the lattice parameters’ determination was 0.05%.

## 3. Results

### 3.1. Temperature Transfer Analysis

Phase transformations in the top layer of the W/Nb system depended on its temperature and heating/cooling time.

The classical equation of heat transfer with the heat energy flux through the surface of the sample was used:

(1) $$\partial T/\partial t=\left(\kappa /c_{eff}\rho \right)\nabla \cdot \left(\nabla T\right),$$

where *κ* is the thermal conductivity (160 W/m·K for W, 55 W/m·K for Nb), *c* is the heat capacity (140 J/kg·K for W, 280 J/kg·K for Nb) and *ρ* is the density of the target (19,350 kg/m^3^ for W, 8570 kg/m^3^ for Nb). The effective value of the heat capacity *c_eff_* was used instead of *c*. This transformation of the heat capacity allowed the heat energy absorption during the phase change to be taken into account [17,18,19]. The equation implies continuous increasing of the temperature for temperatures far from the melting point. In this regard, the heat capacity value is changed as

(2) $$c_{eff}=c+\lambda \delta (T-T_{m}),$$

where *c* is the real heat capacity of the material, *λ* is the latent heat energy for phase transformation and *δ* is the delta function. The delta function *δ* is expressed as a Gauss function:

(3) $$\delta (T-T_{m})=\left(1/\Delta \sqrt{2\pi }\right)\exp\left[-\frac{{\left(T-T_{m}\right)}^{2}}{2\Delta^{2}}\right],$$

where Δ is the temperature range 4K showing the width of the curve.

The energy from the plasma flow was absorbed by the thin layer of the target due to heat conductivity from the plasma state and the solid target. This was taken into account by boundary conditions.

**Figure 1:**
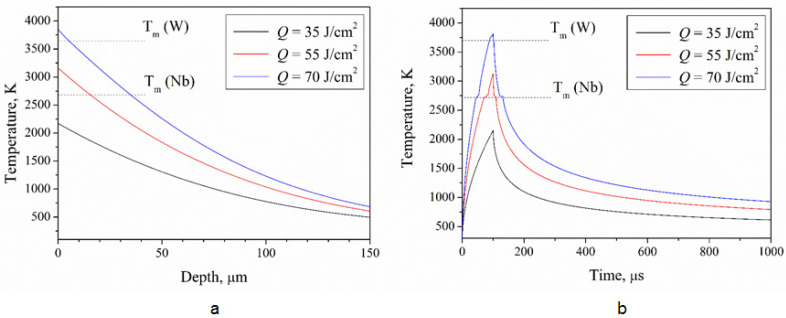
Temperature dependence on: (**a**) depth and (**b**) time in W/Nb system after compression plasma flows’ impact at different absorbed energy densities (Q).

For the simulation of the impact of the Nb/W system heated by the plasma stream with the heat energy of 35–70 J/cm^2^, the temperature on the surface was highest at the end of the plasma pulse, i.e., after 100 μs of the pulse beginning (Figure 1b). The temperature profiles over the depth in the Nb/W system after the CPF impact at the absorbed energy density of 35–70 J/cm^2^ are presented in Figure 1a.

**Figure 2:**
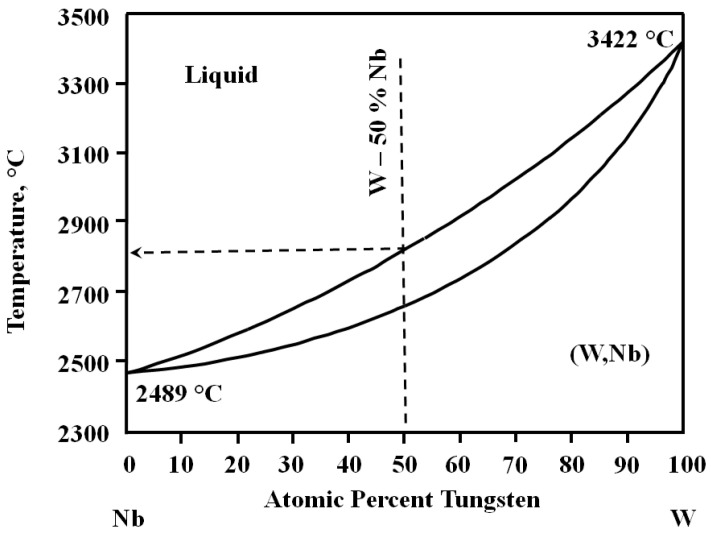
Binary phase diagram of WNb system [20].

Melting of the niobium coating started from the surface at 2740 K and propagated inside the coating. The absorbed energy density of about 35 J/cm^2^ was not enough to melt the niobium coating. Melting of the tungsten substrate should start from the tungsten melting point, 3690 K. However, the interface between the tungsten substrate and the niobium coating can be considered as a thin layer with equiatomic concentration of both elements. The increase in the absorbed energy density up to 55 J/cm^2^ resulted in the niobium coating melting without tungsten. The absorbed energy density close to 70 J/cm^2^ melted both the niobium and tungsten together. The results were obtained without assuming a chemical interaction between the tungsten and niobium. According to the binary phase diagram for tungsten–niobium (Figure 2) [20], the WNb alloy with equiatomic composition melts in the range from 2600 to 2800 K.

Melting of the substrate can be achieved at the absorbed energy lower than that obtained by the simulation. The results obtained show that any metal in the top layer with a lower melting point than the substrate decreased the critical energy required for melting. The melted layer in the interface allowed niobium atoms to diffuse into the solid tungsten phase and decrease its melting point.

Another feature of the pulsed plasma impacts is the high values of the dynamical thermal parameters in the melted state such as the temperature gradient and cooling rate (Table 1), which were determined from the temperature profiles.

A high temperature gradient at the beginning of the crystallization process causes internal mechanical stress in the solid phase. After dissipation of the plasma stream, the heat energy stops transferring to the metal and the molten metal cools rapidly. The cooling rate reached the highest value 2.3 × 10^8^ K/s at the absorbed energy density of 70 J/cm^2^.

### 3.2. Structure and Phase Composition

**Figure 3:**
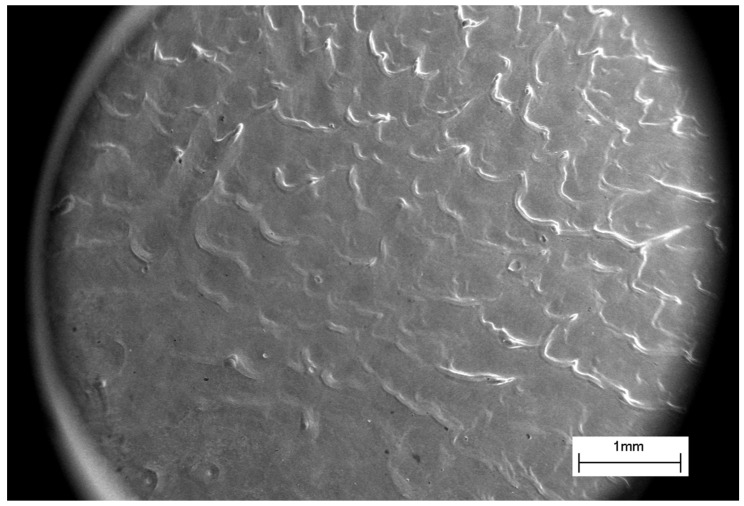
SEM-SE-image of the WNb sample surface after the CPF impact with the absorbed energy density of 70 J/cm^2^.

The WNb sample surface after the CPF exposure with the absorbed energy density of 70 J/cm^2^ is presented in Figure 3 showing that melting and solidification had occurred.

The wavy relief prevailing on the surface of the treated samples formed when molten and indicates hydrodynamic flows along the surface. The observed relief confirms the temperature on the surface exceeded the melting point of both tungsten and niobium after the plasma stream impact, which allowed mixing of the two layers of tungsten and niobium. Figure 3 shows a relatively smooth part at the center of the sample, corresponding to the central part of the plasma flow. The wavy relief was more developed on the edge of the sample and was associated with the plasma flow spreading along the surface. The high viscosity of the tungsten melt combined with hydrodynamic motion resulted in mixing of both the tungsten and niobium melted layers. Such a complex motion of the liquid metals led to the WNb alloy formation on the top of the sample after the CPF impact.

**Figure 4:**
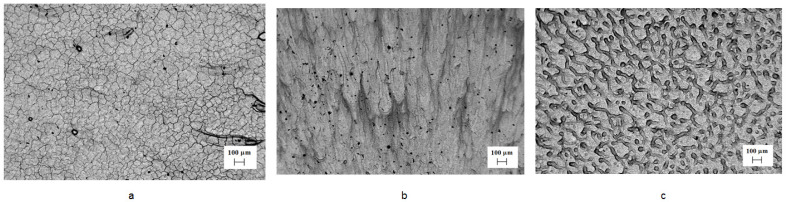
SEM-BSE-images of the WNb alloy’s surface after plasma impact with the absorbed energy densities of (**a**) 70 J/cm^2^, (**b**) 55 J/cm^2^ and (**c**) 35 J/cm^2^.

The tungsten/niobium ratio in the formed WNb alloy was varied by changing the absorbed energy density of the plasma flow and was analyzed by SEM-BSE (Figure 4). The CPF impact with the highest absorbed energy density of 70 J/cm^2^ on the tungsten with niobium film produced a uniform distribution of both elements in the surface layer (Figure 4a). The mean niobium concentration in the layer was 4.6 (±0.2) at.%.

At a lower absorbed energy density of 55 J/cm^2^, niobium distribution on the surface became less uniform. Figure 4b shows darker and lighter areas with different compositions. However, the mean niobium concentration on the modified surface was 30 ± 0.3 at.%. For the absorbed energy density of the plasma flow 35 J/cm^2^, the difference in contrast in the SEM image was more obvious (Figure 4c). There were light areas enriched in tungsten with the 10–15 at.% of niobium concentration. There were elongated darker regions with the 35–40 at.% of niobium concentration. The niobium-enriched areas were directed from the center of the samples towards the edge that is associated with plasma spreading and liquid niobium motion.

There were also cracks after solidification and the cooling solid phase due to the high temperature gradient and cooling rate. Indeed, according to the heat transfer modeling, the temperature gradient near the top layer reaches 10^7^ K/m, while the cooling rate is 10^8^ K/s (Table 1).

**Figure 5:**
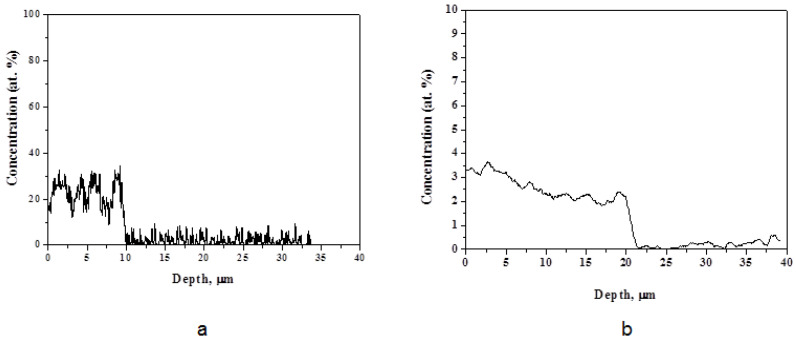
Niobium concentration profile with depth in the WNb alloy formed by the CPF impact with the absorbed energy densities of (**a**) 35 J/cm^2^ and (**b**) 70 J/cm^2^.

The depth distributions of niobium depending on the absorbed energy density were analyzed in cross-sections of the samples using SEM-EDX. The niobium concentration profiles are presented in Figure 5.

The results show the depth of the layer with niobium atoms was about 10 μm after the CPF treatment with the absorbed energy density of 35 J/cm^2^ (Figure 5a). On the surface, there was a nonuniform distribution of niobium. The layers with a niobium concentration of 30 at.% alternated with the layers with 15–20 at.% of the niobium content. The thickness in such sublayers was 2–3 µm. The appearance of such areas resulted from insufficient time of the molten state of both the tungsten and niobium metals that made the mixing ineffective. When the absorbed energy density increased to 70 J/cm^2^, the niobium concentration decreased to 4.0 ± 0.2 at.%; however, the depth of the melted and alloyed layer increased to 20 μm (Figure 5b). Here, a more uniform distribution of niobium over the depth was observed, where it gradually decreased to 2.0 ± 0.2 at.%. Such a sufficiently deep melted layer in tungsten was achieved at the high absorbed energy density from the plasma flow.

**Figure 6:**
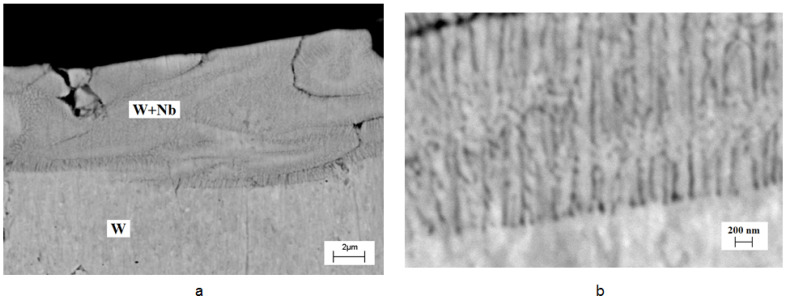
SEM-BSE images of the top layer cross-section for the WNb alloy formed by the CPF impact with the absorbed energy density of 70 J/cm^2^ (different magnifications).

The SEM images of the cross-sectional structure of the WNb alloys produced by the CPF impact show fine columnar growth (Figure 6). The darker contrast of the near-surface layer in the SEM-BSE mode indicated the presence of a lighter atomic number Nb layer (Figure 6a). The WNb binary phase diagram [20] (Figure 2) shows that niobium lowers the melting point of the alloy. Therefore, the niobium-enriched melt had a longer existence time, with the regions without niobium being solidified first. As the heat conductivity of the solid phase is higher than that of the liquid phase, the solid phase of tungsten transferred the heat energy more intensively than the liquid phase and provided directed crystallization.

**Figure 7:**
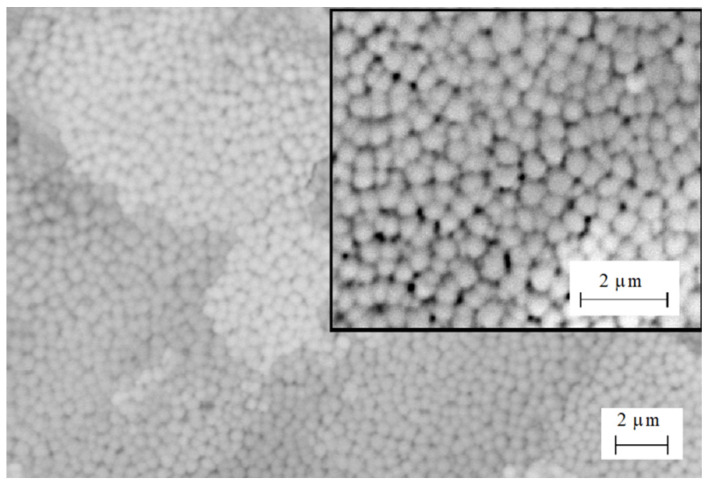
SEM-BSE image of the grain structure on the surface of the WNb alloy formed by the compression plasma flows’ impact at the absorbed energy density 70 J/cm^2^.

The SEM image (Figure 6b) obtained on the boundary between the unmelted tungsten and the melt clearly shows the growth of columnar grains or dendritic structures perpendicular to the interface. The columnar grains grew as a result of the formation of a cellular structure after directional solidification. The average thickness of the columns was 50–100 nm. The chaotic tungsten enriched areas appear in the melt because of insufficient mixing. Fine grains were revealed on the surface of the tungsten in the area where niobium atoms had been detected (Figure 7).

The mean size of the cells was about 300 ± 50 nm. The appearance of the cells on the surface as well as thin columns in the cross-section structure resulted from the instability of the moving crystallization front.

**Figure 8:**
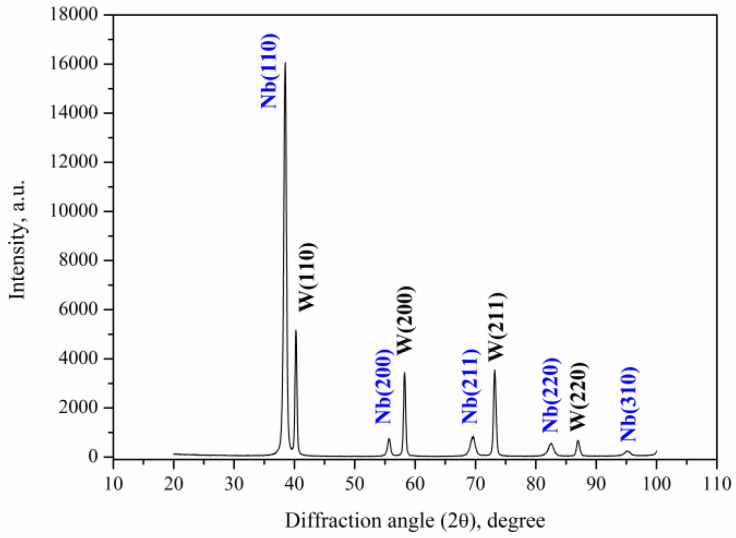
XRD pattern of the Nb/W system in the initial state.

Figure 8 shows the X-ray pattern of the initial state of the sample, which clearly shows a set of the diffraction peaks of both polycrystalline phases: tungsten (JCPDS no. 00-004-0806) and niobium (JCPDS no. 00-034-0370). The diffraction peaks of the tungsten were quite narrow, which indicated a high degree of crystallinity. At the same time, the diffraction peaks of the niobium were wider, which was associated with both a high number of defects during the coating growth by the vacuum arc method and (WNb) solid solution growth.

**Figure 9:**
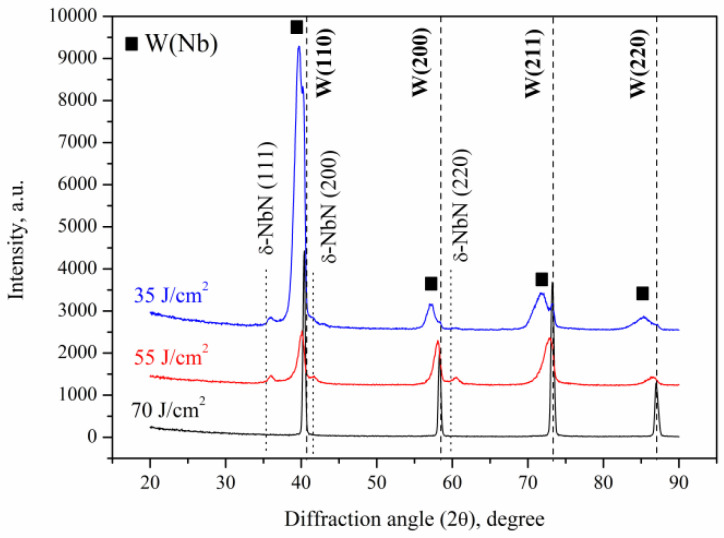
XRD patterns of the WNb alloys formed after three pulses of the CPF impact at different absorbed energy densities.

The diffraction peaks of the niobium phase disappeared after the plasma treatment (Figure 9).

The niobium as a separate phase disappeared, and EDX showed that it dissolved in the W crystal lattice and formed a solid solution. The phase composition of the modified tungsten was controlled by the niobium concentration as well as the absorbed energy density during the plasma exposure. Thus, at the highest absorbed energy density of 70 J/cm^2^, quite narrow diffraction peaks of tungsten were observed. Their angular positions in the XRD pattern completely correspond to the positions of a pure tungsten. The calculated lattice parameter of the bcc tungsten lattice was 0.3165 nm, which corresponds to the standard value 0.3165 nm.

**Figure 10:**
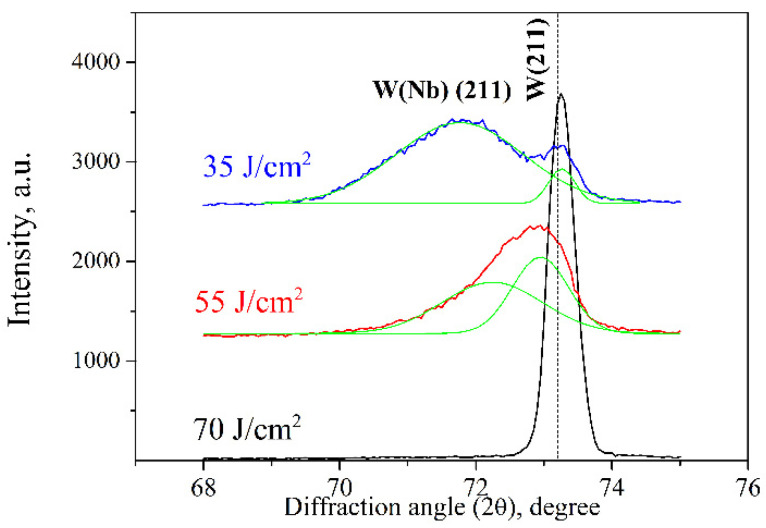
Deconvolution of W and W(Nb) solid solution diffraction lines.

When decreasing the absorbed energy density of the plasma flow to 55 J/cm^2^, the amount of evaporated niobium atoms fell too, and the Nb concentration in the modified layer increased (Figure 9). The position of the diffraction lines shifted towards the smaller angles, indicating an increased lattice parameter of the crystal lattice. A similar shift was also observed in the WNb alloy formed by the plasma flow impact with the absorbed energy density of 35 J/cm^2^. However, in the last case, the diffraction lines broadened. Deconvolution of the broad diffraction lines as two Gaussians (with Origin 2023 software) allowed the superposition of two lines corresponding to two different phases to be found (Figure 10).

Both diffraction patterns show the presence of relatively narrow diffraction peaks on the right side, which can be associated with the phase of pure tungsten without any niobium atoms. Indeed, the lattice parameter equaled 0.3175 nm, which is slightly higher than that of the standard value. The second line on the side of the smaller diffraction angles is characterized by a large FWHM (full width at half-maximum), and corresponds to the bcc phase with the lattice parameter of 0.3202 nm. This broadened diffraction line can be associated with the niobium solid solution phase based on the tungsten lattice W(Nb). According to the phase diagram, tungsten and niobium can dissolve in each other in the solid state in unlimited quantities. Since the main alloying mechanism is connected to the liquid-phase mixing of two liquid metals, the distribution of the niobium atoms over a depth of the modified layer was not uniform and that was especially pronounced in alloys treated with the plasma flow at the lower absorbed energy density of 35 J/cm^2^. Indeed, the diffraction line of the W(Nb) solid solution in this processing regime is characterized by a rather large width due to the inhomogeneity of the composition. Since niobium and tungsten belong to the groups of transition metals with very dense electron shells, the electronic structure of the outer electron shells is believed to be sufficiently stable during the solid solution formation and the atomic radii can be used to explain the change in lattice parameters. When comparing the atomic radii of niobium (145 pm) and tungsten (141 pm) [21], the lattice parameter of the W(Nb) solid solution increased with niobium concentration. The lattice parameter of the W(Nb) solid solution formed by the plasma impact with the absorbed energy density of 35 J/cm^2^ increased to 0.3222 nm due to the rising niobium concentration.

**Figure 11:**
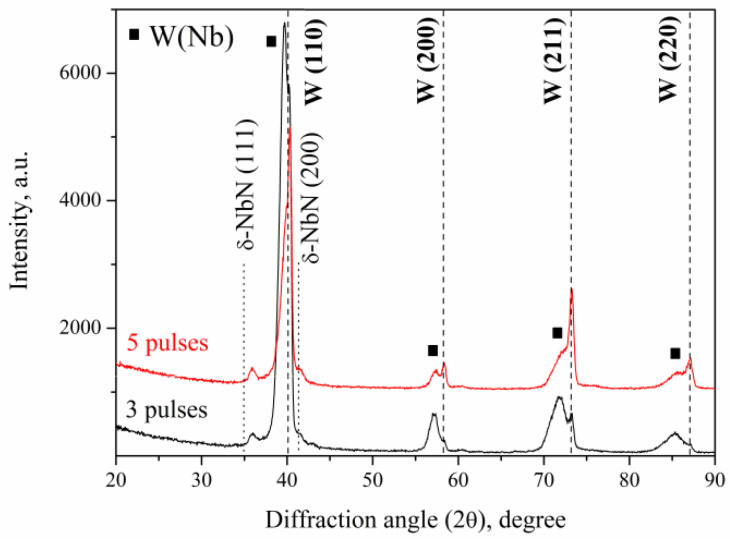
XRD patterns of the WNb alloys formed after three and five pulses of the CPF impact at absorbed energy density of 35 J/cm^2^.

Another way for niobium concentration to change is the pulse number variation. This effect was confirmed by the plasma impact with the lower value of the absorbed energy density of 35 J/cm^2^. The XRD patterns (Figure 11) show both pure tungsten W underneath the modified layer and solid solution W(Nb) phase formation.

The increase in the pulse number up to five shots made the pure tungsten diffraction lines more intensive, indicating the decrease in the volume fraction of the W(Nb) solid solution. It results from a decrease in the total niobium concentration, as niobium was removed after each shot due to evaporation and erosion. Since the niobium concentration decreased after five pulses of plasma stream impacts, the lattice parameter of the W(Nb) solid solution decreased from 0.3222 to 0.3204 nm.

## 4. Discussion

The process of plasma stream interaction with a metal surface determines the change in the surface morphology of the target. After the discharge in the plasma accelerator, the stream of plasma moves towards the surface of the tungsten sample. Being lighter particles, the electrons have a higher velocity compared to the ions and the electron wave reaches the surface first evaporating the thin surface layer from the niobium coating. After this, the evaporated products, such as neutral atoms and ions of niobium, are held together near the surface due to the high pressure of the plasma stream. This compressed gas state of the evaporated atoms is the shock-compressed layer, which plays the role of a transition area between the plasma stream and the surface of the treated sample. It is the shock-compressed layer that conducts the heat energy from the plasma stream to the metal. In this regard, the main part of the energy of the plasma stream transfers to heating the surface of the tungsten. Due to heat conductivity in the solid state, the maximum temperature is achieved on the surface and decreases in the depth. During the total time of the plasma stream impact of 100 μs, the temperature of the surface grows.

The method of alloys synthesis based on the pulsed high-energy impact such as compression plasma flows [10,11], electron beams [22] and ion beams [23] is widely described elsewhere. In the Nb/W system, niobium has a lower melting point than tungsten, so the formation of the liquid phase began in the coating at the absorbed energy density of 55 J/cm^2^. In the energy range from 55 to 67 J/cm^2^, only the niobium coating melted without the tungsten substrate. Therefore, such modes of the pulsed impact are not appropriate for the WNb alloy synthesis. At the energy of 67 J/cm^2^, the temperature at the Nb/W interface reached 3000 K and the tungsten substrate began to melt together with the coating. If tungsten melting occurs at a temperature of (3690 K), the required energy density should be equal to 67 J/cm^2^.

The time of existence of the melt at the absorbed energy density of 55 J/cm^2^ was very short, not more than a few microseconds, as the melting point of the niobium coating was reached at the end of the plasma pulse. When the plasma impact on the Nb/W system had an energy density of 55 J/cm^2^, the niobium coating started to melt at the time of about 85 μs. The crystallization of the melted state totally finished at 105 μs, so the total time of liquid phase existence was about 15–30 μs. This time was estimated as the difference between the time of the beginning and ending of crystallization. The growth of the energy up to 70 J/cm^2^ increased the melting time to 70 μs.

The life-time of the melt in the tungsten–niobium system affected the element distribution on the sample surface. The life-time of the melt produced by the CPF impact with the absorbed energy density of 70 J/cm^2^ was 30 µs, whereas it fell to 15 µs with a decreased absorbed energy density of 55 J/cm^2^. The latter time was not enough for effective mixing of tungsten and niobium, so the structure of the solidified layer was similar to a composite. The niobium was likely to be collected in separate local jets due to its viscosity and surface tension. According to the temperature modeling (Table 1), the absorbed energy density of the plasma flow of 55 J/cm^2^ with a pulse duration of 100 μs was the boundary value for niobium melting. However, the experimental results showed the melting process even at the absorbed energy density of 35 J/cm^2^. The niobium can significantly decrease the melting point of tungsten melt.

Since the tungsten has a high brittle–ductile transition temperature (300–400 °C), the surface layer is brittle and destroyed after cooling to the room temperature. It should be noted that the absorbed energy density influenced the crack density on the surface. The plasma treatment with 70 J/cm^2^ provided the highest crack density when the mean distance between the cracks was about 100 μm. It is the plasma mode that results in the lowest niobium concentration in the top layer.

The presented SEM images show that cracks originate at a depth and propagate towards the surface. The shape of the cracks follows the shape of the areas enriched with tungsten. The crack formation was caused by the presence of nucleation sites at a certain depth where the crystallization front moves from different sides until the moment of their collision. However, due to the smaller specific volume of the solid phase compared to the liquid phase, at the point of collision of the crystallization fronts, there is a lack of a liquid phase for the continuous growth of the solid phase. Thus, a local decrease in the density of the solid phase forms cracks. Therefore, it can be assumed that if it were possible to achieve more uniform mixing of the two components of tungsten and niobium, then this would lead to the absence of bulk crystallization centers and would form a crystallization front from the boundary of the unmelted part to the surface, without cracks. Another reason for the cracks was the difference in Young’s moduli of tungsten (350–400 GPa) and niobium (90–160 GPa). During cooling of the modified layer, the stresses arising due to linear expansion in both phases are different.

Since the plasma stream was formed in the nitrogen residual atmosphere, the interaction between the nitrogen atoms resulted in the nitride phases’ growth. The XRD patterns show the presence of niobium nitride phase δ-NbN with a cubic crystal lattice. Although the formation of tungsten nitride phases was reported [24], it required a sufficiently long time of nitriding, but no tungsten-based nitride phases were found, and no nitride phases were found in the sample after the CPF treatment at the absorbed energy density of 70 J/cm^2^ where the lowest Nb concentration had been found. As the nitrogen saturation of the surface layer occurs due to a diffusion transport mechanism for a short time after the plasma pulse, the nitride phase is formed with a low concentration of nitrogen. It is the main reason for the decrease in the lattice parameter of the δ-NbN phase to 0.4322 nm, which is lower than that for the standard value (0.4394 nm) (JCPDS no. 01-071-0162).

## 5. Conclusions

The presented results showed the possibility of WNb alloy synthesis with high-energy pulsed plasma stream influence on tungsten with 2 μm Nb coating. The main results are as follows:

1. The plasma treatment melted the niobium coating and a part of the tungsten substrate that made it possible to mix both liquid layers and produce a WNb alloy.
2. The composition of the alloy was determined by the relation between the thicknesses of the melted layers and, as a result, by the absorbed energy density. The plasma flow with the absorbed energy density in the range of 35–55 J/cm^2^ predominantly melted the niobium coating and produced the solid solution W(Nb) in the areas in contact with the tungsten. The lattice parameter of the bcc solid solution W(Nb) depended on the niobium concentration.
3. The thickness of the WNb alloy layer increased from 10 to 20 μm with the absorbed energy density rising from 55 to 70 J/cm^2^. The highest absorbed energy density of 70 J/cm^2^ provided a uniform niobium distribution in the modified layer with the niobium concentration of 4 at.%.
4. The plasma treatment with the absorbed energy density from 55 to 70 J/cm^2^ can be effectively used for solid solution W(Nb) production in tungsten. This mode of the treatment provides melting of the top layer without the intense evaporation of niobium.

## Figures and Tables

**Table 1:** Dynamical thermal parameters of the WNb melt after one pulse of the compression plasma flow.

Q, J/cm^2^	Temperature Gradient Near the Surface (*dT*/*dx*), 10^7^ K/m	Cooling Rate Near the SURFACE (*dT*/*dt*), 10^8^ K/s
35	2.1	1.2
55	3.2	2.0
70	4.2	2.3

## Data Availability

The authors declare that the data supporting this study are available from the corresponding author upon request.
